# Gelatin-Based Hydrogel Functionalized with Dopamine and Layered Double Hydroxide for Wound Healing

**DOI:** 10.3390/gels10050318

**Published:** 2024-05-07

**Authors:** Weijie Zhang, Bing Zhang, Yihu Wang, Xiaofeng Cao, Jianing Wang, Weipeng Lu, Yanchuan Guo

**Affiliations:** 1 Key Laboratory of Photochemical Conversion and Optoelectronic Materials, Technical Institute of Physics and Chemistry, Chinese Academy of Sciences, Beijing 100190, China; zhangweijie@mail.ipc.ac.cn (W.Z.); wyh8632@mail.ipc.ac.cn (Y.W.); xfcao@mail.ipc.ac.cn (X.C.); wangjianing@mail.ipc.ac.cn (J.W.); luweipeng@mail.ipc.ac.cn (W.L.); 2 School of Chemical Sciences, University of Chinese Academy of Sciences, Beijing 100049, China

**Keywords:** gelatin, dopamine, LDH, hydrogel, wound healing

## Abstract

Hydrogels with adhesion properties and a wetted structure are promising alternatives to traditional wound dressing materials. The insufficiency of gelatin hydrogels in terms of their adhesive and mechanical strength limits their application in wound dressings. This work presents the design and preparation of a gelatin-based hydrogel functionalized with dopamine (DA) and layered double hydroxide (LDH). The combination of DA and LDH improves the hydrogel’s adhesion properties in terms of interfacial adhesion and inner cohesion. Hydrogels with 8% DA and 4% LDH attained the highest adhesion strength of 266.5 kPa, which increased to 295.5 and 343.3 kPa after hydrophobically modifying the gelatin with octanoyl and decanoyl aldehydes, respectively. The gelatin-based hydrogels also demonstrated a macroporous structure, excellent biocompatibility, and a good anti-inflammatory effect. The developed hydrogels accelerated wound healing in Sprague Dawley rat skin full-thickness wound models.

## 1. Introduction

As the largest organ in the human body, the skin functions as a protective barrier against external invasion [1]. However, skin damage from accidents, surgeries, and burns weakens the barrier, resulting in bleeding that causes inflammation and health hazards [2]. Traditional wound dressing materials such as bandages and gauze [3,4] are usually dry and lack adhesion properties, which means they are not suitable for wound healing, even though they have been widely used clinically. Hydrogels with adhesion properties and a wetted structure are an alternative to traditional wound dressing materials. The continuous and interconnected porous structure of hydrogels is similar to an extracellular matrix, a suitable microenvironment for tissue repair [5,6]. The moist and soft surface of hydrogels could prevent a secondary injury due to friction [7,8]. Several hydrogels have been reported in the literature, but they all have limitations [9]. Developing an ideal adhesive hydrogel for tissue repair is still a significant challenge.

Gelatin has unique physicochemical properties and excellent biocompatibility as a protein extracted from mammalian skin, bone, and connective tissue [10]. Gelatin hydrogels have been widely applied in tissue engineering, wound healing, and drug delivery as they have proven to be beneficial for cell adhesion, migration, and proliferation [11]. Moreover, gelatin can be readily cross-linked or modified with other functional groups or materials to improve its mechanical and chemical properties [12]. The hydrophobic modification of gelatin was reported to increase its adhesive ability due to hydrophobic interactions between molecules in cell membranes and added hydrophobic groups [13].

Hydrogels incorporated with dopamine (DA) could have strong wet tissue adhesion strength by introducing catechol groups. The improved adhesion usually comes from three sources. The first is the self-polymerization of DA [14]. The second is that the catechol group could covalently react via Michael-type addition or Schiff base formation with nucleophile groups on the tissue surface, such as amino and thiol groups, under alkaline or oxidative conditions [15]. The third is that the catechol group could form an immediate interaction with polyvalent metal ions such as Fe^3+^ [16].

Layered double hydroxide (LDH) is an anionic clay material consisting of an outer layer of positively charged metal hydroxide and an interlayer of negatively charged inorganic or organic anions [17]. Due to its layered structure, ion exchange capacity, and good biocompatibility [18], LDH has been researched for applications in biomedical and bioengineering. LDH is conducive to wound healing by providing an alkaline environment, while human fibroblasts proliferate in an alkaline environment with a pH of 8.5–9.5 [19]. On the other hand, the existence of LDH in hydrogels could enhance its mechanical properties by promoting polymer chain entanglement.

**Scheme 1:**
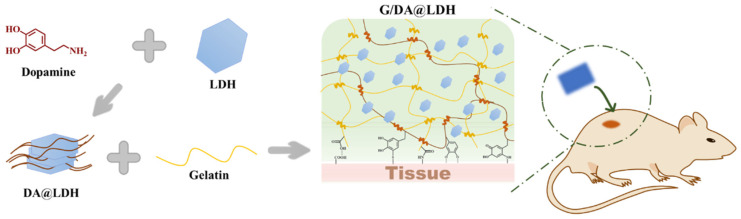
Schematic representation of the formation of the G/DA@LDH.

This work develops gelatin-based hydrogels functionalized with DA and LDH and hydrophobically modified gelatin with octanoyl and decanoyl aldehydes to enhance their adhesive properties. As shown in Scheme 1, DA intercalated into the LDH and cross-linked with the gelatin. The adhesion of the hydrophobically modified gelatin-based hydrogels comes from the adhesive, cohesive, and hydrophobic interactions. The physicochemical properties of the hydrogels were investigated through a series of characterizations, and the cytocompatibilities and anti-inflammatory effects were explored to consider the biocompatibilities in vitro. The wound closure effects of the hydrogels were evaluated using a Sprague Dawley (SD) rat skin full-thickness wound model in vivo.

## 2. Results and Discussion

### 2.1. Synthesis and Characterization of LDH and DA@LDH

**Figure 1:**
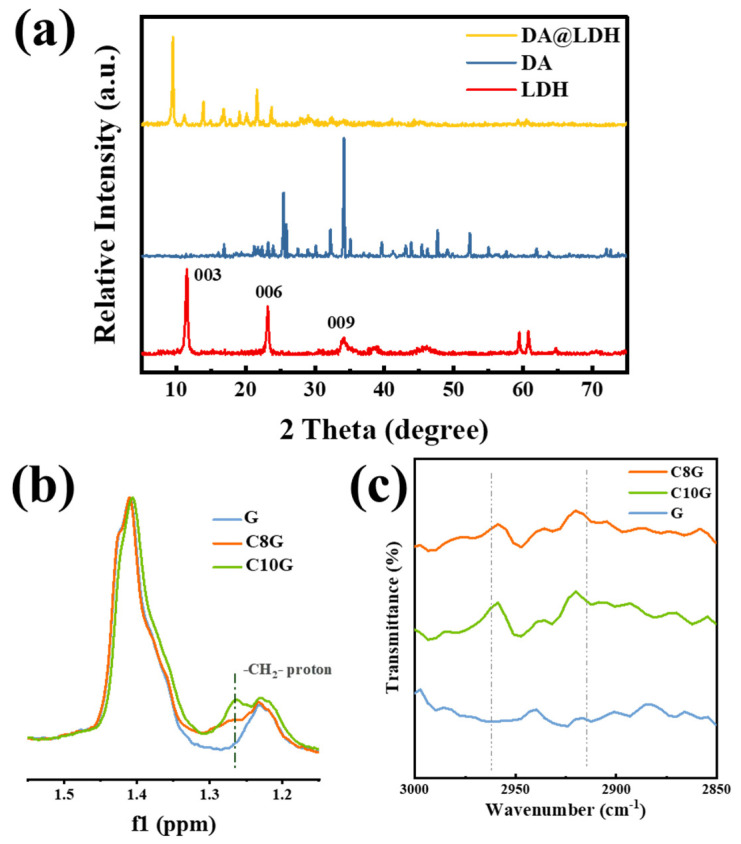
(**a**) XRD patterns of the DA, LDH, and DA@LDH; (**b**) ^1^H-NMR spectra, and (**c**) FT-IR spectra of the G, C8G, and C10G.

The inorganic component, LDH, was synthesized and added into the hydrogels to enhance its energy dissipation and improve the mechanical strength. The reaction in Scheme 1 shows the nanostructured DA@LDH composite with conformal surfaces synthesized via the intercalative polymerization of DA in a metal hydroxide interlayer [20]. The color of DA@LDH changed to black from the white DA because the hydroxyl group of LDH facilitates the oxidation reaction. The form of the polydopamine (PDA) from DA was synthesized by the following process: DA was first converted to p-benzoquinone via electron transfer, then to DHI (5,6-dihydroxyindole)–DHI dimers and DA-DHI-DHI trimers through covalent oxidization, and finally to PDA via oxidization and self-assembly. The X-ray diffraction (XRD) patterns of the LDH and DA@LDH are shown in Figure 1a. The LDH displayed sharp and symmetrical (00*l*) diffraction peaks at 2*θ* = 11.5° and 23.2°, presenting a relatively good and complete crystal layered structure, indicating the successful synthesis of the LDH layered structure [18]. Similar (00*l*) diffraction peaks at 2*θ* = 9.49° and 21.6° were also observed for the DA@LDH. The basal spacing of the DA@LDH increased to 9.31 and 4.11 Å based on Bragg’s Law [21], corresponding to (003) and (006) reflections, respectively, from 7.69 and 3.83 Å for the LDH. The increased basal spacing demonstrates structural deformation, confirming that the self-polymerized PDA was intercalated into the LDH layers. According to Nam et al. [20], the π-stacked structure of the PDA had an interlayer spacing of approximately 3.4–3.8 Å, lower than the basal spacing of LDH. Thus, it could intercalate into the layer space of the LDH.

### 2.2. Synthesis and Characterization of Hydrophobically Modified Gelatin

Hydrophobically modified gelatin samples were synthesized by reductive amination between amino groups in the gelatin (G) and aldehyde groups in the aliphatic aldehydes in the presence of 2-picoline borane [22] to enhance the interfacial strength. The properties of the hydrophobically modified gelatin with different alkyl chain lengths (C6, C8, and C10) and modification ratios (10%, 15%, and 20%) were measured, as shown in Figure S1. The water contact angle, gel strength, and viscosity of the hydrophobically modified gelatin increased with the alkyl chain length and modification ratio. The octanoyl-modified gelatin (C8G) and decanoyl-modified gelatin (C10G), with modification ratios near 15%, could be applied to synthesize adhesive hydrogels because of their suitable viscosity and hydrophobic properties. The modification ratios of C8G and C10G were 14.7 mol% and 14.4%, respectively, as calculated by the residual amino group content determined by the 2,4,6-trinitrobenzensulfonic acid (TNBS) method, as shown in Table 1. The hydrophobic modification of C8G and C10G were also confirmed by the FT-IR and ^1^H-NMR spectra in Figure 1b,c. The methylene groups were added in G after modification. In the FT-IR spectra, the characteristic -CH_2_- stretching peaks of C8G and C10G were significantly enhanced at 2947 and 2932 cm^−1^. The protons of -CH_2_- were at 1.27 ppm in the ^1^H-NMR spectra [23]. Peaks at 1.27 ppm were observed in the ^1^H-NMR spectra for both C8G and C10G, and the peak intensity of C10G was stronger. These results indicated that hydrophobically modified gelatins, C8G and C10G, were successfully synthesized.

### 2.3. Synthesis and Physicochemical Characterization of Hydrogels

**Figure 2:**
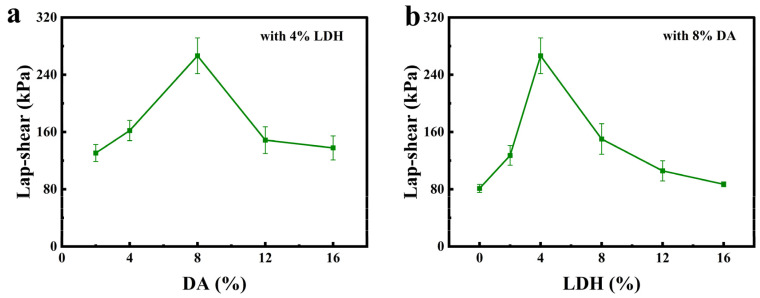
Lap-shear strength of gelatin-based hydrogels with different concentrations of (**a**) DA and (**b**) LDH.

Two factors could affect the adhesion properties of the gelatin-based hydrogels: interfacial adhesion and inner cohesion. The interfacial adhesion of hydrogels is related to the concentration of DA, as shown in Figure 2a. The hydrogels reached the best adhesion when the DA content was 8%. Inner cohesion is largely affected by LDH concentration, as shown in Figure 2b. The adhesion property increased with the LDH content. The LDH facilitates the orientation of the polymer chains in the hydrogel to resist external stress, realized through the increased lap-shear strength [24]. The hydrogel with a 4% LDH content had the greatest lap-shear strength. The adhesion strength decreased if the LDH content was increased further. The distance between the LDH molecules decreased when there was excessive LDH content. The limited distance restricts the sliding and ductility of the polymer chains between the LDH layers [25]. Therefore, the suitable DA and LDH concentrations for their incorporation in the hydrogels are 8% and 4%, respectively.

**Figure 3:**
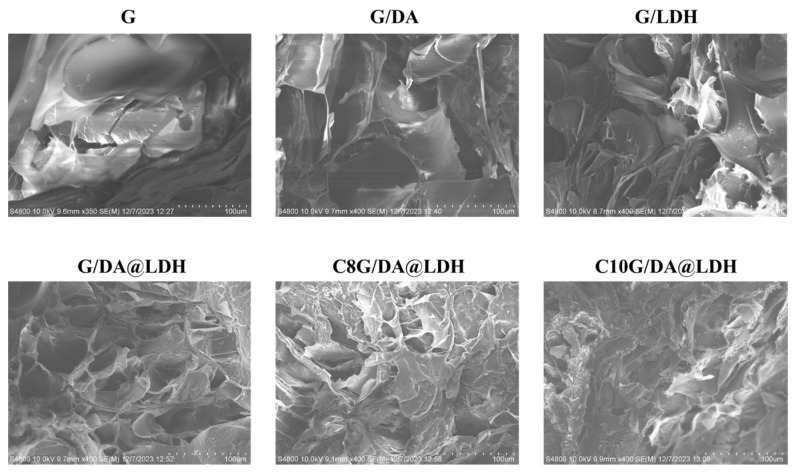
SEM images of the created hydrogels.

The microstructures of the hydrogel samples, observed via scanning electron microscopy (SEM), are shown in Figure 3. Like the hydrogels from [26,27], the gelatin-based hydrogel samples all displayed an interconnected and well-proportioned macroporous network. This structure is similar to the extracellular matrix and is beneficial for wound healing [28,29]. After adding LDH, it was homogeneously dispersed in the skeleton of the hydrogels, and the resulting rougher surface contributed to adhesion and cell growth. The supportive effect of LDH also leads to a denser network with a slight decrease in pore size. The aliphatic chain introduced by hydrophobic modification increased the cross-linking between hydrogels, further decreasing the pore size. The surface functional groups of the hydrogels were determined via Fourier-transform infrared (FT-IR) spectroscopy, as shown in Figure S2. The -OH stretching peak at 3550–3200 cm^−1^ was observed in all samples because of the -OH group. The chemical bonding of DA and gelatin was demonstrated as the peak of the CO-NH bond at 1640 cm^−1^.

### 2.4. Physicochemical Properties of Hydrogels

#### 2.4.1. Swelling Property

**Figure 4:**
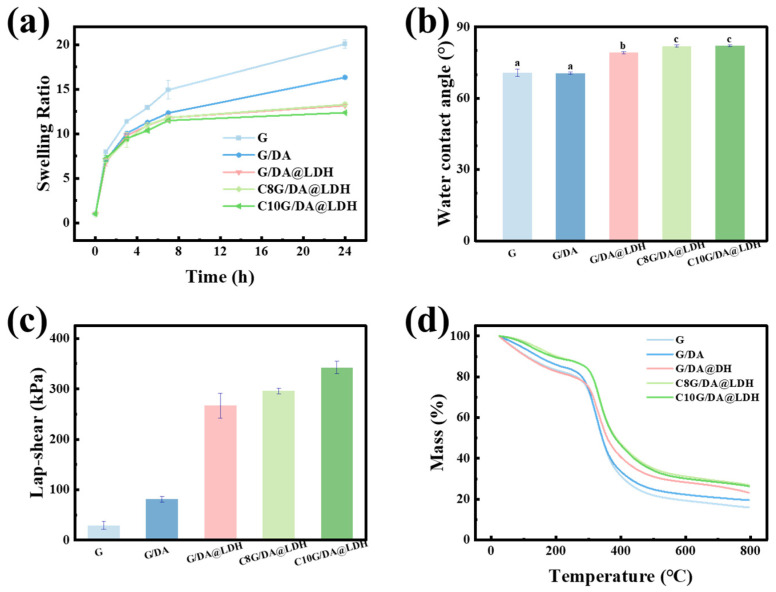
(**a**) Swelling ratios, (**b**) water contact angles, (**c**) adhesion, and (**d**) thermogravimetric graph of hydrogels. Different superscripts in the same column indicate significant differences (*p* < 0.05).

The swelling properties of hydrogels are essential for absorbing tissue exudation and maintaining adhesive stability [26]. These property are also an indicator of hydrogels’ cross-linking degree [11]. The swelling curves of the samples are shown in Figure 4a, indicating the hydrogels almost reached swelling equilibrium after 24 h. The G sample had the highest swelling ratio over 24 h. The swelling ratio declined with the addition of DA and LDH due to the increased cross-linking density. C8G/DA@LDH showed no difference in swelling ratio with G/DA@LDH, while C10G/DA@LDH displayed a slight decrease. The results are in accordance with the ^1^H-NMR spectra, with a more obvious ethyl proton peak in C10G. Therein, the inorganic content and hydrophobic modification both lead to a more cross-linked and more stable hydrogel structure. This prevents high water absorption, decreasing resistance to the interfacial strength between tissue and hydrogel [23].

#### 2.4.2. Water Contact Angle

The water contact angles were measured to evaluate the wettability of the hydrogels (Figure 4b). The water contact angles of G and G/DA were 70.7° and 70.5°, respectively. After incorporating LDH, the wettability of G/DA@LDH was altered significantly, with a water contact angle of 79.1°. The water contact angle increased with the alkyl chain length in the hydrophobically modified gelatin. Thus, C8G/DA@LDH and C10G/DA@LDH displayed a more hydrophobic surface, with water contact angles of 81.7° and 82.1°, respectively. The results agree well with the adhesive strength of the hydrogels.

#### 2.4.3. Adhesive Strength

The adhesion of the gelatin-based adhesive hydrogels was determined via lap-shear tests between gelatin-coated PET sheets to simulate the surfaces of tissue and organs. As shown in Figure 4c, G had the smallest adhesion. With the addition of DA, the mussel-inspired functional group catechol was introduced, and the G/DA adhesion increased. Incorporating LDH into the hydrogel greatly increased the adhesion of G/DA@LDH. The adhesion of hydrogels relies on the two aspects of the adhesive strength between hydrogels and tissues and the cohesive strength within the hydrogels [30]. The DA group in G/DA@LDH enhanced the adhesive strength from Micheal addition and Schiff base reaction with tissue, as shown in Scheme 1. The LDH content promoted the cohesive strength. The presence of LDH improved the body strength of the hydrogel, enhancing the cohesive energy and adhesive strength [9].

The gelatin was hydrophobically modified to further improve adhesion under wet conditions. The hydrophobic surfaces of C8G/DA@LDH and C10G/DA@LDH enhance the affinity of hydrophobic molecules in tissues and organs, such as fibronectin and elastin [13]. On the other hand, the hydrophobic chains in C8G/DA@LDH and C10G/DA@LDH break the hydration layer on the tissue surface, and the resulting nucleophile groups of tissues are exposed to the catechol groups of the hydrogels, increasing the adhesion between hydrogels and tissues. The adhesive strength results agreed well with our expectations, and the hydrophobically modified hydrogels showed the greatest adhesive strength.

#### 2.4.4. Thermal Stability

The thermal gravimetric analysis (TGA) curves of the gelatin-based hydrogels are shown in Figure 4d. All samples displayed two weight loss periods under a N_2_ atmosphere. The first weight loss phase occurred below 300 °C due to the evaporation of water that had been physically absorbed and combined between layers. The weight loss was proportional to the swelling ratio of the hydrogels. The second weight loss phase is attributed to the degradation of the organic groups in the hydrogels. By the end of the heating process, G/DA@LDH had undergone a weight loss of 76.9%, while C8G/DA@LDH and C10G/DA@LDH lost 73.1% and 73.7%, respectively. The gelatin-based hydrogels had remarkably higher thermal stability than G and G/DA. After hydrophobic modification, the thermal stabilities of C8G/DA@LDH and C10G/DA@LDH were slightly greater than that of G/DA@LDH.

### 2.5. Biocompatibility of Hydrogels

**Figure 5:**
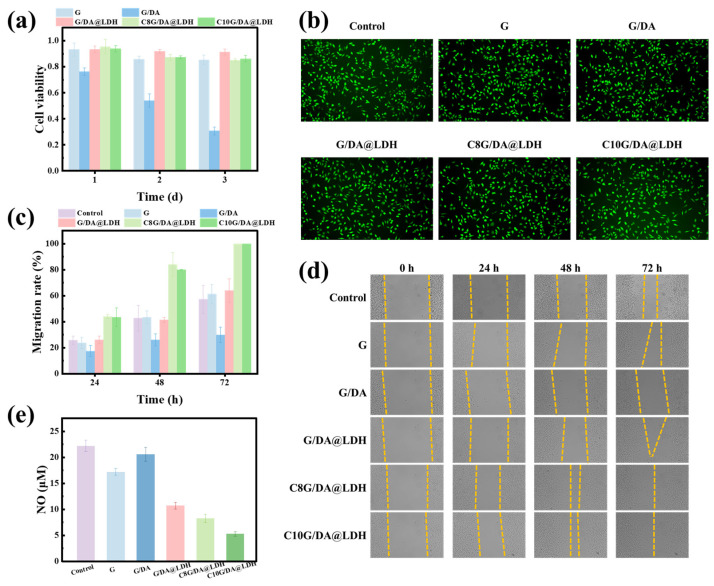
(**a**) Cell viability, (**b**) live/dead staining, (**c**,**d**) cell migration, and (**e**) anti-inflammatory effects of hydrogels.

Hydrogels’ excellent biocompatibility is essential for their biological application. CCK-8 assays were performed using the L929 cell line to evaluate the in vitro cytotoxicity of the gelatin-based hydrogels, as shown in Figure 5a. Only G/DA showed slight toxicity. The potential toxicity of the catechol group might be the cause, and it is released in greater amounts due to its looser structure compared to LDH-modified hydrogels [31]. G/DA@LDH, C8G/DA@LDH, and C10G/DA@LDH displayed no significant differences in the cell viability of the L929 cells cultured in the hydrogel extracts, and the cell viabilities were all above 85% after 1, 2, and 3 days of co-culturing. This indicates that the gelatin-based hydrogels G/DA@LDH, C8G/DA@LDH, and C10G/DA@LDH had good cytocompatibility. This was also verified by live/dead staining assays (Figure 5b). The L929 cells were alive and spaced regularly with a spindle-like morphology after 1 day of incubation. There were barely any dead cells observed. Cell scratch assays were performed to explore the effect of the hydrogels on cell migration, as demonstrated in Figure 5c,d.

The migration rates increased, and the scratch area decreased over time in all hydrogels. The migration rate of the G/DA was significantly lower than the control group, which could be caused by its toxicity to the cells. The hydrophobically modified gelatin-based hydrogels displayed remarkably higher migration rates, implying that the hydrophobic modification contributed to cell migration and was beneficial for wound healing.

The wound healing process includes several periods: inflammation, tissue formation, and tissue remodeling [32]. Anti-inflammatory activity is another important requirement for hydrogel dressings. The production of NO by RAW264.7 macrophage cells is an indicator of inflammation after some stimulation. The anti-inflammatory effects of the hydrogels were investigated by measuring the level of NO after co-culturing with LPS and the hydrogel extracts for 24 h. Figure 5e shows that the gelatin-based hydrogels significantly suppressed NO production in the RAW264.7 cells. The decrease was over 50%, which increased for the hydrophobically modified gelatin-based hydrogels to 63% and 76%, respectively.

### 2.6. Wound Closure Evaluation of Hydrogels

**Figure 6:**
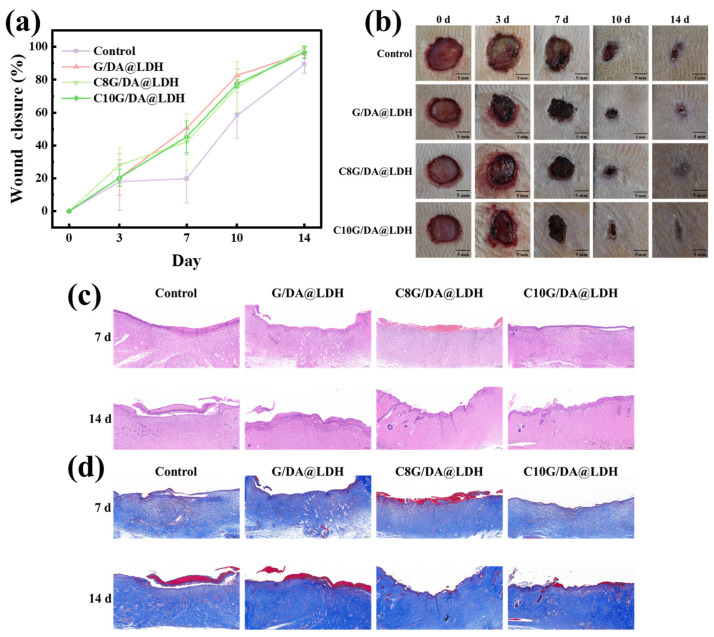
(**a**,**b**) Wound closure abilities of hydrogels; (**c**) H&E and (**d**) Masson’s trichrome staining images of a wound.

The wound closure effects of the hydrogels were evaluated using an SD rat skin full-thickness wound model. The gelatin-based hydrogels could closely adhere to the skin and seal defects well. The healing speeds of the gelatin-based hydrogels were all faster than the control group, as shown in Figure 6a,b, and the wound defects of the gelatin-based hydrogels scabbed within 7 days, faster than the control group. After 14 days, the wounds in the gelatin-based hydrogel groups were almost completely healed, similar to and even better than those in references [27,33,34]. As demonstrated in the H&E staining pictures in Figure 6c, there was less inflammatory cell infiltration in the gelatin-based hydrogels, agreeing with the in vitro experiments. On day 7 (early stage of wound healing), there were fewer collagen fibers in all groups, while on day 14 (last stage of wound healing), there were more collagen fibers deposited (Figure 6d). The arrangement of collagen fibers was more orderly in the gelatin-based hydrogel groups, as the fibers were irregular and swirling in the control group. The keratinized layers of the gelatin-based hydrogels were much thicker than those of the control group, and the hydrophobic modifications promoted the formation of the keratinized layers. In summary, the gelatin-based hydrogels could accelerate the repair of wound defects.

## 3. Conclusions

Mussel-inspired gelatin-based hydrogels were developed and prepared for wound treatment based on the enhancements in the interfacial adhesion from DA, the cohesive strength from LDH, and the hydrophobic effect from hydrophobically modified gelatin. The hydrophobically modified gelatin-based hydrogels displayed a macroporous network, good adhesive ability, suitable swelling properties, excellent biocompatibility, and good anti-inflammatory properties. The gelatin-based hydrogels accelerated wound healing in the full-thickness wound model in vivo. It is expected that gelatin-based hydrogels will be a potential clinical adjuvant treatment in the future.

## 4. Materials and Methods

### 4.1. Materials

Hydrochloric acid, sodium hydroxide, ethanol, Mg(NO_3_)_2_·6H_2_O, and Fe(NO_3_)_3_·9H_2_O were purchased from Pecking Reagent (Beijing, China). Dopamine hydrochloride (DA) was purchased from Aladdin Scientific Corp (Shanghai, Chinia). Gelatin was provided by Baotou Dongbao Bio-tech Co., Ltd. (Baotou, China). Octyl aldehyde, decyl aldehyde, and 2-picoline borane were purchased from Shanghai Macklin Biochemical Co., Ltd. (Shanghai, China).

### 4.2. Synthesis of LDH

The MgFe-LDH (LDH) was prepared by separate nucleation and aging steps [35]. The Mg(NO_3_) _2_·6H_2_O and Fe(NO_3_) _3_·9H_2_O and the NaOH and Na_2_CO_3_ were separately dissolved in 100 mL of water. The two solutions were simultaneously added to a colloid mill rotating at 3000 rpm and mixed for 1 min. The resulting slurry was moved to a flask and aged at 80 °C for 24 h. The final precipitate was filtered and washed. The LDH was dried at 60 °C after being washed successively with water and ethanol. The X-ray diffraction (XRD) patterns of the samples were obtained using a Bruker DAVINCI D8 ADVANCE diffractometer with a scan range of 5–75°.

### 4.3. Synthesis of Hydrophobically Modified Gelatins

The octanoyl-modified gelatin (C8G) and decanoyl-modified gelatin (C10G) were prepared by the reductive amination of amino groups in gelatin with hexyl aldehyde and octyl aldehyde [23]. Then, 30 g of gelatin was dissolved in 105 mL of a water/ethanol mixture at 55 °C. A 0.15 eq of hexyl aldehyde or octyl aldehyde was added to the amino group of the gelatin and stirred for 1 h. The 2-picoline borane was then added to the mixture, and the solution was stirred overnight at 55 °C. Precipitates were produced by adding a large volume of ethanol. The hydrophobically modified gelatin was dried at 60 °C after being washed with ethanol. The modification ratios of C8G and C10G were determined by measuring the residual amino group content using TNBS as described previously [36]. The modifications to the C8 and C10 groups in the hydrophobically modified gelatin were confirmed by Fourier-transform infrared (FT-IR) spectroscopy and ^1^H-NMR.

### 4.4. Preparation of Gelatin-Based Hydrogels

#### 4.4.1. Synthesis of DA@LDH

A total of 0.04 g of LDH was immersed in 10 mL of water and evenly dispersed via ultrasound for 1 h. Then, 0.08 g of DA was added into the suspension to obtain DA@LDH after oxidation under magnetic stirring at room temperature for 24 h.

#### 4.4.2. Preparation of Hydrogels

Hydrogels with compositions based on gelatin (G/DA@LDH), C8G (C8G/DA@LDH), and C10G (C10G/DA@LDH) were fabricated through a simple process. A total of 1.0 g of gelatin, C8G, or C10G was dissolved into the reacted DA@LDH suspension at 65 °C and cooled to obtain a gelatin-based hydrogel.

### 4.5. Physicochemical Properties of Hydrogels

The gelatin-based hydrogels were characterized by several physical and chemical techniques. Their morphologies were analyzed via scanning electron microscopy (SEM, S4800, HITACHI, Tokyo, Japan). Their FT-IR spectra were determined using an Excalibur 3100 instrument (Varian, Palo Alto, CA, USA) with a scanning range from 4000 to 400 cm^−1^ at room temperature. Thermal gravimetric analysis (TGA) was performed using a simultaneous thermal analyzer (Q50, TA instruments, New Castle, DE, USA) under a N_2_ atmosphere from 20 to 800 °C at a heating rate of 10 °C/min. The water contact angles were measured using an automatic contact angle meter (OCA-20, DataPhysics, Filderstadt, Germany) after dropping a 3 μL droplet of ultra-pure water on the hydrogels. The hydrogels were lyophilized before the swelling experiments, and the swelling behaviors were determined by testing their water uptake capacity. The hydrogels were incubated in PBS for 24 h, and their wet weights were determined at 0, 1, 3, 5, 7, and 24 h. The swelling ratio was calculated according to the following equation:

(1) $$Swelling\;ratio=\frac{W-W_{0}}{W_{0}}$$

where W is the wet weight of the hydrogels, and $W_{0}$ is the dry weight.

The lap-shear tests were performed to test the adhesive abilities of the hydrogels using gelatin-coated PET sheets for simulating the tissue surface, according to modified ASTM F2255 protocols [37]. A CT3 texture analyzer (Brookfeild CO., Middleboro, MA, USA) equipped with a 4000 g load cell was used to determine the adhesion strength of the gelatin-based hydrogels. The adhesive area was 20 mm × 10 mm, and the tensile rate was 13 mm/min.

### 4.6. Biocompatibility of Hydrogels

#### 4.6.1. Cytotoxicity Testing

CCK8 colorimetry and live/dead cell staining were used to explore the cytotoxicity of the hydrogels [38]. L929 cells were evenly distributed on a 96-well plate and cultured in DMEM containing 10% FBS at 5% CO_2_ concentration at 37 °C for 24 h. Then, the mediums were replaced with 100 μL of 10 mg/mL hydrogel extracts and co-cultured for 1, 2, and 3 days, with DMEM used as the control group. CCK-8 was added to each wall, and absorbance was measured at 450 nm. Cell viability was calculated using the following formula:

(2) $$Cell\;viability=\frac{A_{sample}-A_{blank}}{A_{control}-A_{blank}}$$

where $A_{blank}$, $A_{control}$, and $A_{sample}$ represent the absorbance values of the blank, control, and sample groups at 450 nm. The cells’ morphologies were observed via live/dead staining [9]. After co-culturing for 1 d with hydrogel extracts, the L929 cells were stained with PI and Calcein-AM dye and observed under a fluorescent microscope (DMI6000 B, Leica, Solms, Germany).

#### 4.6.2. Cell Migration Assay

Cell migration testing was conducted using L929 cells. The L929 cells were cultured in DMEM with 10% FBS in a 6-well plate until their confluence reached 80%. The cell debris was washed with PBS after making straight scratches. The L929 cells were co-cultured with 2 mL of 10 mg/mL hydrogel extracts, and the scratched areas were observed and photographed under a microscope. The migration rate (%) was calculated as follows:

(3) $$Migration\;rate\;\left(\%\right)=\frac{S_{t}}{S_{0}}\times 100\%$$

where $S_{t}$ and $S_{0}$ are the scratched areas at *t* h and 0 h, respectively.

#### 4.6.3. Anti-Inflammatory Property

The RAW264.7 macrophages were plated into 48-well plates and cultured in DMEM with 10% FBS at a 5% CO_2_ concentration until their confluence reached 80%. The medium was replaced with 10 mg/mL hydrogel extracts, and RAW264.7 macrophages were stimulated with 100 ng/mL of LPS for 24 h. The endogenously generated NO concentration was determined using a Griess reagent kit. DMEM incorporating 100 ng/mL LPS was used as a positive control group.

### 4.7. In Vivo Wound Healing Evaluation

All animal experiments were performed following the guidelines of the National Regulation of China for Care and Use of Laboratory Animals after obtaining permission from the Ethics Committee. Male Sprague Dawley (SD) rats were acclimatized for a week, then anesthetized, and their back hair was shaved. Full-thickness wounds with diameters of about 10 mm were made on the shaved area. The wounds were divided into control (PBS), G/DA@LDH, C8G/DA@LDH, and C10G/DA@LDH groups. The wound closure areas were observed and photographed on days 0, 3, 7, and 14. The wound areas were measured using Image J software, and wound closures were calculated according to the following formula:

(4) $$Wound\;closure\;\left(\%\right)=\left(1-\frac{W_{t}}{W_{0}}\right)\times 100\%$$

where $W_{t}$ and $W_{0}$ are the wound areas on *t* d and 0 d, respectively. The regenerated tissues were collected and immersed in 4% formaldehyde. The tissue sections were stained with hematoxylin–eosin (H&E) reagents and Masson based on the manufacturer’s instructions [39]. All slices were analyzed and photographed using a microscope.

### 4.8. Statistical Analysis

Statistical analyses were performed using SPSS software (version 28.0). Data are presented herein as means ± standard deviations. The statistical significance between the results was determined by a one-way analysis of variance (ANOVA).

## Figures and Tables

**Table 1:** Modification ratios of hydrophobically modified gelatins.

Sample	Aliphatic Aldehydes Added (mol%)	Modification Ratio (mol%)
C8G	15	14.7 ± 2.5
C10G	15	14.4 ± 1.3

## Data Availability

The original contributions of the study are included in the article. Further inquiries can be directed to the corresponding authors.

## Supplementary Materials

The following supporting information can be downloaded at: https://www.mdpi.com/article/10.3390/gels10050318/s1. Figure S1: (a) Water contact angle, (b) viscosity, (c) gel strength, and (d) melting point of hydrophobically modified gelatin. Figure S2: FT-IR spectra of hydrogels.
