# Supplementary material for: Gelatin-Based Hydrogel Functionalized with Dopamine and Layered Double Hydroxide for Wound Healing
Source: Gels. 2024 May 7;10(5):318. doi: 10.3390/gels10050318 (PMC11120944; doi:10.3390/gels10050318)
Supplement: Supplementary file 1 [file gels-10-00318-s001.zip › gels-2970205-supplementary.pdf]

Supporting Information

Gelatin-based hydrogel functionalized with dopamine and LDH for wound healing

Weijie Zhang[1,2], Bing Zhang[1,*], Yihu Wang[1], Xiaofeng Cao[1], Jianing Wang[1], Weipeng Lu[1], and Yanchuan Guo[1,2,*]

[1] Key Laboratory of Photochemical Conversion and Optoelectronic Materials, Technical Institute of Physics and Chemistry, Chinese Academy of Sciences, Beijing 100190, China

[2] School of Chemical Sciences, University of Chinese Academy of Sciences, Beijing 100049, China

[*] Corresponding author: zhangbing@mail.ipc.ac.cn; yanchuanguo@mail.ipc.ac.cn

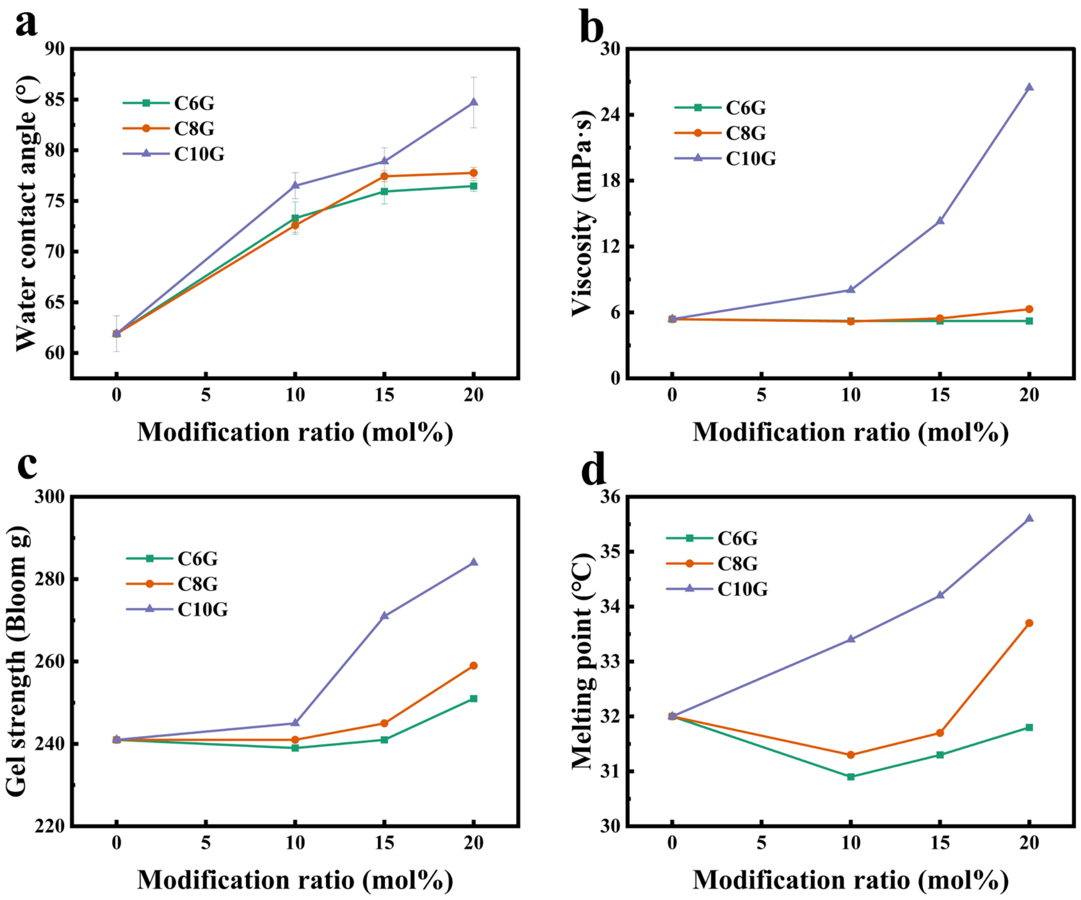

Figure S1. (a) Water contact angle, (b) viscosity, (c) gel strength, and (d) melting point of hydrophobically modified gelatin

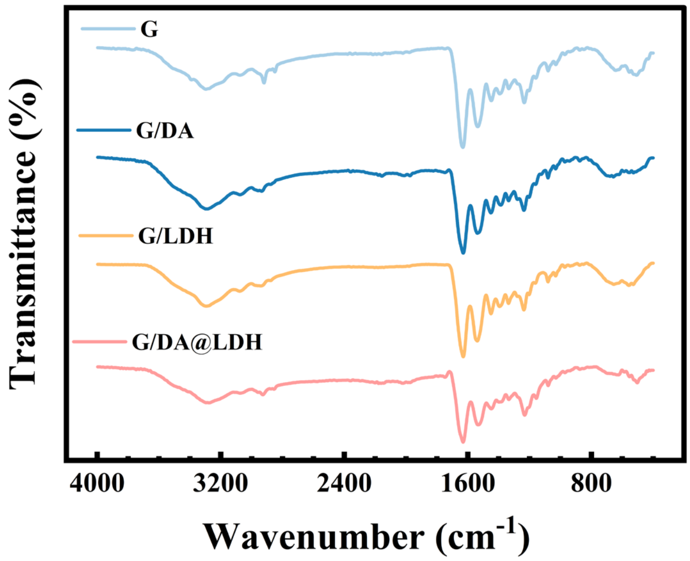

Figure S2. FT-IR spectra of hydrogels
